# Applying the intention-to-treat principle in practice: Guidance on handling randomisation errors

**DOI:** 10.1177/1740774515588097

**Published:** 2015-08

**Authors:** Lisa N Yelland, Thomas R Sullivan, Merryn Voysey, Katherine J Lee, Jonathan A Cook, Andrew B Forbes

**Affiliations:** 1 Women’s & Children’s Health Research Institute, The University of Adelaide, Adelaide, SA, Australia; 2 School of Population Health, The University of Adelaide, Adelaide, SA, Australia; 3 Nuffield Department of Primary Care Health Sciences, University of Oxford, Oxford, UK; 4 Murdoch Children’s Research Institute, Parkville, VIC, Australia; 5 Department of Paediatrics, University of Melbourne, Melbourne, VIC, Australia; 6 Centre for Statistics in Medicine, Nuffield Department of Orthopaedics, Rheumatology and Musculoskeletal Sciences, University of Oxford, Oxford, UK; 7 Surgical Intervention Trials Unit, University of Oxford, Oxford, UK; 8 Department of Epidemiology and Preventive Medicine, Monash University, Melbourne, VIC, Australia

**Keywords:** Randomisation error, intention to treat, guidelines, randomised controlled trial, ineligible participant

## Abstract

**Background:**

The intention-to-treat principle states that all randomised participants should be analysed in their randomised group. The implications of this principle are widely discussed in relation to the analysis, but have received limited attention in the context of handling errors that occur during the randomisation process. The aims of this article are to (1) demonstrate the potential pitfalls of attempting to correct randomisation errors and (2) provide guidance on handling common randomisation errors when they are discovered that maintains the goals of the intention-to-treat principle.

**Methods:**

The potential pitfalls of attempting to correct randomisation errors are demonstrated and guidance on handling common errors is provided, using examples from our own experiences.

**Results:**

We illustrate the problems that can occur when attempts are made to correct randomisation errors and argue that documenting, rather than correcting these errors, is most consistent with the intention-to-treat principle. When a participant is randomised using incorrect baseline information, we recommend accepting the randomisation but recording the correct baseline data. If ineligible participants are inadvertently randomised, we advocate keeping them in the trial and collecting all relevant data but seeking clinical input to determine their appropriate course of management, unless they can be excluded in an objective and unbiased manner. When multiple randomisations are performed in error for the same participant, we suggest retaining the initial randomisation and either disregarding the second randomisation if only one set of data will be obtained for the participant, or retaining the second randomisation otherwise. When participants are issued the incorrect treatment at the time of randomisation, we propose documenting the treatment received and seeking clinical input regarding the ongoing treatment of the participant.

**Conclusion:**

Randomisation errors are almost inevitable and should be reported in trial publications. The intention-to-treat principle is useful for guiding responses to randomisation errors when they are discovered.

## Introduction

The intention-to-treat (ITT) principle is widely discussed in the randomised trials literature.^1–13^ While the exact definition of ITT is a matter of considerable debate, particularly in the presence of missing outcome data,^6,8^ ITT is generally taken to mean that all randomised participants should be analysed in their randomised group, irrespective of compliance with the trial protocol. An ITT analysis is widely accepted as the gold standard for assessing the superiority of the intervention in randomised trials. It preserves the balance in prognostic factors achieved by randomisation, which is important for avoiding selection bias and establishing causation.^2^ A popular alternative is to conduct a ‘modified ITT analysis’, which has been used to describe a variety of deviations from the standard ITT approach, such as exclusion of participants who were found to be ineligible or failed to receive any treatment.^10^

Although rarely recognised, the ITT principle has implications for dealing with errors that occur during the randomisation process. These include participants being randomised using incorrect baseline information, ineligible participants being randomised, multiple randomisations being performed for the same participant, and participants receiving the incorrect treatment following randomisation. We refer to such errors as ‘randomisation errors’. By considering how participants affected by randomisation errors should be handled in an ITT analysis, researchers can respond to these errors when they are discovered in a way that will minimise their impact on the analysis and the need to deviate from ITT.

While randomisation errors are almost inevitable, guidance on handling these errors is limited,^14^ and the application of the ITT principle in this context has not been considered to our knowledge. The aims of this article are to (1) demonstrate the potential pitfalls of attempting to correct randomisation errors and (2) provide guidance on handling common randomisation errors when they are discovered. In developing our recommendations, we sought to maintain the goals of the ITT principle by minimising the potential for bias in the treatment effect estimate. In this article, we take the ITT principle to mean that all randomised participants are included in the analysis in their randomised group. Examples of randomisation errors are drawn from our experiences as trial statisticians, investigators and members of independent data monitoring committees involved mainly with publicly funded randomised trials in health-related disciplines.

In what follows, we make several assumptions about the underlying trial and randomisation process. First, all participants are assigned a unique identifier (a study ID), and a corresponding treatment group during randomisation. Second, this information is stored in an electronic randomisation record along with the date and time of randomisation, and any baseline variables used in the randomisation process. Third, the errors occur in the implementation of the randomisation schedule, rather than its design. Fourth, the randomisation process is automated using a computer or telephone randomisation service. Manual randomisation processes are prone to additional problems^15,16^ that are not considered here. Finally, the primary purpose of the trial is to assess the superiority of an intervention, and hence, an ITT analysis is of interest.

## Methods and results

### Potential pitfalls of attempting to correct randomisation errors

Requests for randomisation errors to be ‘fixed’, by removing any record of the incorrect randomisation and proceeding as if it never occurred for example, are common in our experience. Although these requests may seem sensible, attempts to fix randomisation errors often make the problem worse.^14^ We provide two case studies that demonstrate the potential pitfalls of trying to correct randomisation errors and make general recommendations regarding such corrections. Guidance on handling specific randomisation errors is given later in this article.

#### Case study 1

In a placebo controlled trial, consenting participants were assigned the next available study ID and corresponding treatment allocation from the randomisation schedule and issued a bottle pre-labelled with their study ID that contained either active or inactive capsules, as appropriate. A participant assigned study ID 3113 was mistakenly given the bottle labelled 3133. The error was discovered several weeks after treatment had commenced, when a new participant was assigned study ID 3133 and the corresponding bottle could not be located. A staff member attempted to fix the error by giving the second participant the bottle labelled 3113. Unfortunately, the affected study IDs were associated with different treatment allocations and the switching of study IDs made the problem worse, since both participants received the incorrect treatment. Such crossover between treatment groups is problematic, as it can reduce the estimated treatment effect under an ITT analysis.^2^ Had the staff member reported the error when it was discovered, a new bottle containing capsules from the correct treatment group could have been prepared for the second participant, and only the first participant would have received the incorrect treatment.

#### Case study 2

In a blinded trial employing stratified randomisation with randomly permuted blocks, a participant belonging to stratum A was incorrectly assigned the next available study ID (8014) and corresponding treatment group from stratum B. Before the participant received any treatment, a staff member reported the error and requested the incorrect randomisation be undone by releasing study ID 8014 to be reassigned to the next participant belonging to stratum B, allowing the affected participant to be re-randomised correctly in stratum A. By the time this request was received by the coordinating centre, another participant had been randomised in stratum B. This raised questions about what should be done with study ID 8014 if released as suggested. Reallocating this ID to the next participant randomised in stratum B would cause the randomisations in this stratum to become non-sequential, making the randomisation records difficult to audit. Conversely, skipping study ID 8014 would create a gap in the randomisation schedule, interfering with the blocking and potentially introducing imbalance in the number of participants randomised to each group. In either case, a systematic deviation from the intended randomisation sequence would occur, which was deemed unlikely to introduce bias but could harm trial credibility. Had the trial been open-label, reallocating study ID 8014 would have been even more problematic due to the lack of allocation concealment following the initial randomisation. Fortunately, the trial investigators decided to maintain the initial randomisation for the participant, thus avoiding any further problems due to the initial error.

#### General recommendations

While it may be possible to fix randomisation errors by undoing the incorrect randomisation, especially if discovered and reported immediately, we caution against this for several reasons. First, our case studies clearly demonstrate the potential for corrections to cause further problems. Second, attempting to correct randomisation errors often violates the ITT principle, since all randomised participants should be analysed according to their initial randomisation, whether or not the randomisation was performed as intended. Finally, allowing randomisation errors to be undone introduces the possibility of selection bias, since participants assigned to the less favourable treatment in an open-label trial, or responding poorly in a blinded trial, may be examined more closely for randomisation errors due to desire for reallocation. We therefore agree with previous recommendations to document but not correct randomisation errors,^14^ as this is the safest approach and is most consistent with the ITT principle.

### Guidance on handling common randomisation errors when discovered

Responding to randomisation errors can be challenging. General recommendations, such as documenting but not correcting randomisation errors, can help guide responses. However, the statistical, clinical, ethical and practical issues associated with the error should be considered before an appropriate course of action is chosen, and compromises are often required. We provide recommendations for handling common randomisation errors when they are discovered, as summarised in Table 1.

**Table 1.** Summary of recommendations for handling common randomisation errors when they are discovered.

Randomisation error	Recommendations
Participant randomised using incorrect baseline information.	Accept the randomisation but record the correct baseline information.
Ineligible participant randomised.	Keep the participant in the trial and collect all relevant data, unless an unbiased process for excluding ineligible participants has been pre-specified. Seek clinical input to determine their appropriate management.
Participant randomised multiple times: (a) Only one set of baseline and outcome data will be obtained. (b) Multiple sets of baseline and outcome data will be obtained.	(a) Retain the initial randomisation and disregard the second randomisation. (b) Retain both randomisations, unless an unbiased process for excluding the second randomisation has been pre-specified.
Participant received incorrect treatment.	Record the treatment the participant received and seek clinical input regarding their ongoing treatment.

#### Participant randomised using incorrect baseline information

Stratified randomisation or minimisation is often used to ensure treatment groups are balanced with respect to specific baseline variables that strongly influence the outcome.^17^ These approaches may fail to achieve balance if randomisations are performed using incorrect baseline information. An example of this was seen in case study 2 and there are several reasons why such errors occur. First, the researcher randomising the participant may receive incorrect baseline information. This was seen in a trial stratified by use of dietary supplements containing a specific nutrient of interest, where a participant was classified in the wrong stratum after providing the incorrect name for the dietary supplement they had been taking. Second, the researcher may have access to the correct baseline information but enter it incorrectly into the randomisation service. This occurred in a trial stratified by age, where the date of birth was incorrectly entered for a participant and their automatically calculated age placed them in the incorrect stratum. Baseline information errors may be recognised immediately or discovered later through reviewing the randomisation records and cross-checking against an alternate data source.

When participants are randomised according to incorrect baseline information, under the ITT principle they should be analysed in their allocated treatment group, irrespective of the fact that their allocation was based on incorrect information. We therefore recommend that the initial randomisation is accepted and the participant is treated according to their group allocation, rather than re-randomised. The incorrect baseline information should be kept in the randomisation record, as this reflects how the randomisation was actually performed,^14^ and the correct information documented for use in an adjusted analysis.^18,19^

#### Ineligible participant randomised

Participants should only be randomised once their eligibility has been confirmed. Unfortunately, even in the presence of clear inclusion and exclusion criteria, it is not unusual for ineligible participants to be inadvertently randomised. This may result from trial staff receiving inaccurate or incomplete information about potential recruits, or failing to correctly apply all inclusion and exclusion criteria. For example, in a trial enrolling extremely preterm infants, a staff member randomised an ineligible infant after incorrectly calculating their gestational age. Participant ineligibility can be difficult to detect following randomisation, but may be discovered through cross-checking the baseline information against an alternate source or through further examination of randomised participants.

If a randomised participant is found to be ineligible, a decision needs to be made regarding their ongoing management. The allocated treatment must be withheld or stopped if the reason for ineligibility means that commencing or continuing treatment is inappropriate, suboptimal or potentially unsafe. For example, a participant with high baseline potassium levels was inadvertently randomised into a trial where the intervention was contraindicated for such individuals, and hence treatment was ceased when their ineligibility was discovered. In other scenarios, it may be problematic to cease treatment based on personal or clinical reasons. We advocate seeking clinical input when making such decisions.

Whether participants should remain in the trial once their ineligibility is discovered depends on whether they should contribute to the ITT analysis, which is a matter of some debate. While standard application of the ITT principle requires that all randomised participants are included in the analysis, regardless of eligibility, it is possible to exclude ineligible participants from the analysis without biasing the treatment group comparison if the reason for and discovery of the ineligibility is independent of treatment group and outcome. For example, bias is unlikely to be introduced if ineligible participants are excluded by an independent panel, blinded to treatment group, who review eligibility for all participants based on their status before randomisation.^1,2,20^ If exclusions cannot be made in an objective and unbiased manner, then ineligible participants should be included in the analysis.^20^ We therefore recommend that ineligible participants who are incorrectly randomised remain in the trial and have all relevant data collected, unless an unbiased process for excluding ineligible participants from the analysis has been pre-specified, in which case they may be withdrawn. However, we recognise that following this recommendation may not always be appropriate. For example, when a randomised participant was deemed ineligible because they had not provided informed consent, they could not ethically remain in the trial and were thus withdrawn. When ineligible participants are withdrawn, we propose keeping their randomisation record for auditing purposes and noting the reason for the withdrawal.

#### Participant randomised multiple times

In some trials, participants can legitimately be randomised multiple times. For example, in pregnancy trials with long recruitment periods, women may be eligible to participate during more than one pregnancy. More commonly, only a single randomisation is allowed per participant and multiple randomisations constitute a randomisation error. Such errors can arise unintentionally or deliberately due to a perceived problem with the initial randomisation.

When the second randomisation is performed in close succession to the first (on the same day for example), the affected participant typically only has one set of baseline and outcome data, and only receives one treatment. The ITT principle suggests that the affected participant should be analysed in the treatment group assigned during the first randomisation, independent of any subsequent randomisations that occurred in error. We thus propose that participants incorrectly randomised multiple times in close succession retain the study ID and receive the treatment allocated during the initial randomisation. The second randomisation can then be disregarded and the reason documented, but the randomisation record should be kept. This approach allows the affected participant to be included in the analysis according to the first randomisation, which means that all randomised participants can be analysed, in keeping with the ITT principle. Importantly, exclusion of the second randomisation from the analysis is not expected to introduce bias into the treatment effect estimate, provided its discovery is uninfluenced by treatment allocation or outcome. This can be achieved by checking whether any trial participants have the same identifying information. Failure to exclude the second randomisation would result in some participants being included in the analysis twice with the same data, potentially under different randomised groups, which would artificially inflate the sample size.

When there is a substantial time delay between multiple randomisations for the same participant, they may have different baseline or outcome data recorded under each randomisation, and may receive multiple treatments. Since the affected participant is actually ineligible at the time of the second randomisation, recommendations provided for handling ineligible participants can be applied to the second randomisation (i.e. it should only be excluded based on a pre-specified unbiased process), although additional complexities arise here. First, the participant may have already received some treatment under the first randomisation and this could influence decisions regarding their ongoing management. Second, if it is decided that no exclusions will be made during the analysis because of the potential for introducing bias, the non-independence of observations arising from repeated participation in the trial should be considered.

#### Participant received incorrect treatment

Treatment issuing errors can arise when the treatment information provided at the time of randomisation is misread or misinterpreted. In addition to case study 1, this occurred in an open-label trial comparing a device intended to assist patients with a particular medical condition to no device, when a participant received the device after a researcher misread their treatment assignment. These errors may be discovered by cross-checking the randomisation records with the documented treatment received or during product inventory. Treatment issuing errors also occur when the assigned treatment is not available, as sometimes occurs in drug trials when the drug pack labelled with the assigned number cannot be located. These errors would typically be identified immediately.

When treatment issuing errors are discovered, a decision must be made regarding the ongoing treatment of the participant. In a standard ITT analysis, participants will be analysed as part of the allocated treatment group, independent of the treatment they received. Since any treatment effect is diluted when participants receive the incorrect treatment,^2^ participants would ideally switch to the correct treatment when errors are discovered. However, the appropriate course of action depends on how soon the error is discovered, the nature and intended duration of the treatment, whether switching treatments is feasible, and the clinical implications of switching treatments, such as whether a washout period is required. Changing treatments may lead to confusion for the participant and is often inappropriate, for example in surgical trials where the error is discovered after another operation has been performed. Continuing with the incorrect treatment is often the safest and simplest approach. When treatment issuing errors are discovered, we recommend seeking clinical input before making decisions regarding the ongoing treatment of the participant. In blinded trials, decisions should be made blinded to the allocated treatment group to preserve the integrity of the blinding. While no changes should be made to the assigned treatment group in the randomisation record, the treatment the participant actually received should be documented.

## Discussion

We have discussed errors that commonly occur during the randomisation process and provided recommendations on how they should be handled when discovered. In general, we believe the safest option is to accept the randomisation errors that do occur and leave the initial randomisation records unchanged. This approach is consistent with the ITT principle, since it enables participants to be analysed as randomised, and avoids further problems that can arise when attempts are made to correct randomisation errors. A potential disadvantage of accepting randomisation errors is that imbalance could be introduced between the randomised groups in the number of participants or their baseline characteristics. However, any imbalance due to randomisation errors is expected to be minimal unless errors are common. Imbalance can be monitored by an independent data monitoring committee during the trial and investigated by the trial statistician at the analysis stage.

Prevention of randomisation errors is important for maintaining trial quality. Strategies for preventing errors include thoroughly testing the randomisation service before the trial commences; reviewing and confirming the information entered into the randomisation service before each randomisation is performed; specifying clear and simple eligibility criteria and recruitment processes; and requiring two staff members to check the treatment allocation or blinded treatment code before any treatment is issued. Unfortunately, avoiding randomisation errors altogether is often impossible. Individuals responsible for randomising participants should therefore be well trained in all aspects of the randomisation process, including the actions to be taken when errors occur. Randomisation errors should be reported as soon as they are discovered so the details can be documented and the appropriate course of action determined. Decisions regarding errors are best made in consultation with the trial statistician, who can assess the implications of the proposed course of action for the analysis.

While randomisation errors are occasionally documented in trial publications,^21–24^ there is currently little incentive for researchers to report these errors, since it is not a requirement in the widely adopted Consolidated Standards of Reporting Trials (CONSORT) statement.^11,25^ We advocate reporting the number and type of randomisation errors that occurred in the trial along with how they were handled, or stating that no randomisation errors were identified, as this will assist readers to assess trial quality and the success of the randomisation. This practice could be encouraged by including randomisation errors in a future update of the CONSORT statement.

In conclusion, randomisation errors are not uncommon and deserve greater attention in the literature. We hope this article has raised awareness of the types of randomisation errors that can occur and provided useful guidance on dealing with such errors when they are discovered. We encourage researchers to consider the ITT principle when deciding how to handle randomisation errors in their trials.

## References

[1] Fergusson D Aaron SD Guyatt G Post-randomisation exclusions: the intention to treat principle and excluding patients from analysis. BMJ 2002; 325: 652–654. 12242181 10.1136/bmj.325.7365.652 PMC1124168

[2] Heritier SR Gebski VJ Keech AC Inclusion of patients in clinical trial analysis: the intention-to-treat principle. Med J Aust 2003; 179: 438–440. 14558871 10.5694/j.1326-5377.2003.tb05627.x

[3] Polit DF Gillespie BM Intention-to-treat in randomized controlled trials: recommendations for a total trial strategy. Res Nurs Health 2010; 33: 355–368. 20645423 10.1002/nur.20386

[4] Gravel J Opatrny L Shapiro S The intention-to-treat approach in randomized controlled trials: are authors saying what they do and doing what they say? Clin Trials 2007; 4: 350–356. 17848496 10.1177/1740774507081223

[5] Hollis S Campbell F What is meant by intention to treat analysis? Survey of published randomised controlled trials. BMJ 1999; 319: 670–674. 10480822 10.1136/bmj.319.7211.670 PMC28218

[6] Alshurafa M Briel M Akl EA Inconsistent definitions for intention-to-treat in relation to missing outcome data: systematic review of the methods literature. PLoS ONE 2012; 7: e49163. 23166608 10.1371/journal.pone.0049163 PMC3499557

[7] Montori VM Guyatt GH Intention-to-treat principle. CMAJ 2001; 165: 1339–1341. 11760981 PMC81628

[8] White IR Horton NJ Carpenter J Strategy for intention to treat analysis in randomised trials with missing outcome data. BMJ 2011; 342: d40. 21300711 10.1136/bmj.d40 PMC3230114

[9] Detry MA Lewis RJ The intention-to-treat principle: how to assess the true effect of choosing a medical treatment. JAMA 2014; 312: 85–86. 25058221 10.1001/jama.2014.7523

[10] Abraha I Montedori A Modified intention to treat reporting in randomised controlled trials: systematic review. BMJ 2010; 340: c2697. 20547685 10.1136/bmj.c2697 PMC2885592

[11] Moher D Hopewell S Schulz KF CONSORT 2010 explanation and elaboration: updated guidelines for reporting parallel group randomised trials. BMJ 2010; 340: c869. 20332511 10.1136/bmj.c869 PMC2844943

[12] Shrier I Steele RJ Verhagen E Beyond intention to treat: what is the right question? Clin Trials 2014; 11: 28–37. 24096636 10.1177/1740774513504151

[13] Green S Benedetti J Crowley J Clinical trials in oncology. 2nd ed. Boca Raton, FL: Chapman & Hall/CRC, 2003, p. 171.

[14] Downs M Tucker K Christ-Schmidt H Some practical problems in implementing randomization. Clin Trials 2010; 7: 235–245. 20484491 10.1177/1740774510368300

[15] Torgerson DJ Roberts C Understanding controlled trials – randomisation methods: concealment. BMJ 1999; 319: 375–376. 10435967 10.1136/bmj.319.7206.375 PMC1126995

[16] Schulz KF Subverting randomization in controlled trials. JAMA 1995; 274: 1456–1458. 7474192

[17] Kahan BC Morris TP Reporting and analysis of trials using stratified randomisation in leading medical journals: review and reanalysis. BMJ 2012; 345: e5840. 22983531 10.1136/bmj.e5840 PMC3444136

[18] Kahan BC Morris TP Improper analysis of trials randomised using stratified blocks or minimisation. Stat Med 2012; 31: 328–340. 22139891 10.1002/sim.4431

[19] Guiloff RJ Clinical trials in neurology. London; New York: Springer, 2001, p. 139.

[20] Schulz KF Grimes DA Sample size slippages in randomised trials: exclusions and the lost and wayward. Lancet 2002; 359: 781–785. 11888606 10.1016/S0140-6736(02)07882-0

[21] Love RR Duc NB Allred DC Oophorectomy and tamoxifen adjuvant therapy in premenopausal Vietnamese and Chinese women with operable breast cancer. J Clin Oncol 2002; 20: 2559–2566. 12011136 10.1200/JCO.2002.08.169

[22] Beckett NS Peters R Fletcher AE Treatment of hypertension in patients 80 years of age or older. N Engl J Med 2008; 358: 1887–1898. 18378519 10.1056/NEJMoa0801369

[23] MacArthur C Shennan AH May A Effect of low-dose mobile versus traditional epidural techniques on mode of delivery: a randomised controlled trial. Lancet 2001; 358: 19–23. 11454372 10.1016/S0140-6736(00)05251-X

[24] Campbell M Fiddian N Fitzpatrick R The knee arthroplasty trial (KAT) design features, baseline characteristics, and two-year functional outcomes after alternative approaches to knee replacement. J Bone Joint Surg Am 2009; 91: 134–141. 10.2106/JBJS.G.01074 19122088

[25] Schulz KF Altman DG Moher D CONSORT 2010 statement: updated guidelines for reporting parallel group randomised trials. BMJ 2010; 340: c332. 20332509 10.1136/bmj.c332 PMC2844940

